# PSD-95: An Effective Target for Stroke Therapy Using Neuroprotective Peptides

**DOI:** 10.3390/ijms222212585

**Published:** 2021-11-22

**Authors:** Lola Ugalde-Triviño, Margarita Díaz-Guerra

**Affiliations:** Instituto de Investigaciones Biomédicas “Alberto Sols”, Consejo Superior de Investigaciones Científicas-Universidad Autónoma de Madrid (CSIC-UAM), Arturo Duperier 4, 28029 Madrid, Spain; lugalde@iib.uam.es

**Keywords:** AVLV-144, calpain, cell-penetrating peptides, excitotoxicity, ischemia, NA-1, nerinetide, neuroprotection, PSD-95, stroke, TP95_414_

## Abstract

Therapies for stroke have remained elusive in the past despite the great relevance of this pathology. However, recent results have provided strong evidence that postsynaptic density protein-95 (PSD-95) can be exploited as an efficient target for stroke neuroprotection by strategies able to counteract excitotoxicity, a major mechanism of neuronal death after ischemic stroke. This scaffold protein is key to the maintenance of a complex framework of protein interactions established at the postsynaptic density (PSD) of excitatory neurons, relevant to neuronal function and survival. Using cell penetrating peptides (CPPs) as therapeutic tools, two different approaches have been devised and advanced to different levels of clinical development. First, nerinetide (Phase 3) and AVLX-144 (Phase 1) were designed to interfere with the coupling of the ternary complex formed by PSD-95 with GluN2B subunits of the N-methyl-D-aspartate type of glutamate receptors (NMDARs) and neuronal nitric oxide synthase (nNOS). These peptides reduced neurotoxicity derived from NMDAR overactivation, decreased infarct volume and improved neurobehavioral results in different models of ischemic stroke. However, an important caveat to this approach was PSD-95 processing by calpain, a pathological mechanism specifically induced by excitotoxicity that results in a profound alteration of survival signaling. Thus, a third peptide (TP95_414_) has been recently developed to interfere with PSD-95 cleavage and reduce neuronal death, which also improves neurological outcome in a preclinical mouse model of permanent ischemia. Here, we review recent advancements in the development and characterization of PSD-95-targeted CPPs and propose the combination of these two approaches to improve treatment of stroke and other excitotoxicity-associated disorders.

## 1. Introduction

Stroke is the second leading cause of death in the world. In 2019, there were 12.2 million incident cases, 101 million prevalent cases and 6.55 million deaths from stroke (11.6% of total deaths) [1]. It is also the leading cause of acquired neurological disability and the second cause of dementia. Thus, stroke represents a significant health issue that, according to demographic changes, is expected to worsen in the future. 

Significant progress has been made in stroke management in recent years. Pharmacological therapy for the ischemic type (≈85% of total stroke cases) is still limited to thrombolytic drugs (e.g., tissue plasminogen activator or tPA), which can be only administered in a very short therapeutic window and are contraindicated for hemorrhagic stroke and other frequent medical conditions [2,3]. Unfortunately, the diagnostic tests able to discriminate the precise stroke type are mainly brain-imaging techniques, not immediately available to all patients. Thus, tPA is only administered to about 10% of patients. Similarly, endovascular thrombectomy, which mechanically restores blood flow, is restricted to some patients [4].

The evidence indicates the importance of developing efficient neuroprotective therapies for stroke treatment. Nevertheless, in the past, a large number of neuroprotective drugs have been tested preclinically and clinically, but none of them have succeed [5]. These failures have raised concern in the field of neuroprotection and shifted the interest to explore delayed mechanisms of stroke (e.g., plasticity, regeneration), or stroke-associated comorbidities, as possible therapeutic targets [6]. However, recent results obtained by the group led by Dr. Tymianski (University of Toronto, Canada) in a Phase 3 clinical trial strongly suggest that early neuroprotection is feasible in patients suffering from acute ischemic stroke (AIS) [7,8,9]. These exciting results have been obtained using brain-accessible peptides able to inhibit specific functions of postsynaptic density protein-95 (PSD-95) that contribute to excitotoxic signaling, a pathological mechanism central to stroke that is induced by overstimulation of the N-methyl-D-aspartate type of glutamate receptors (NMDARs). Development of additional PSD-95-targeted peptides, based on this same strategy or a complementary one, unveil PSD-95 as a highly promising target for stroke treatment.

## 2. Ischemic Stroke and Excitotoxicity

During ischemic stroke, the occlusion of a blood vessel leads to decreased perfusion of a brain area, followed by deprivation of nutrients and oxygen. Two brain areas can be distinguished in the affected brain, the infarct core and the penumbra. While the infarct core contains irreversibly damaged tissue, the penumbra represents an important target for neuroprotective strategies. This area, which only suffers a moderate blood flow reduction, is functionally impaired but remains metabolically active. Thus, the penumbra can be recovered if the blood flow is restored or the cells become more resistant. However, after the primary damage, the penumbra can also suffer processes of secondary neuronal death that cause a gradual expansion of the infarct core. A fundamental mechanism of this delayed form of neuronal death is excitotoxicity, induced by overactivation of the NMDARs.

### Dual Roles of NMDARs in Neuronal Survival and Death

This family of glutamate receptors are ligand-gated ion channels highly permeable to Ca^2+^. In physiological situations, NMDARs are central to neuronal transmission, synaptic plasticity and memory, as well as being central to survival [10]. They are heterotetramers formed by two obligatory GluN1 subunits and two GluN2 (GluN2A–D) or GluN3 (GluN3A–B) subunits, although the most frequently expressed NMDARs contain GluN1 in combination with either GluN2B or GluN2A subunits. The synaptic NMDARs form large and dynamic signaling complexes in the postsynaptic membrane, mostly by interactions established by their intracellular C-terminal domains with signaling, cytoskeleton and scaffold proteins such as PSD-95 [11], which functions as a postsynaptic density (PSD) organizer. Pro-survival signaling initiated by synaptic NMDAR activation and a moderate Ca^2+^ increase stimulates, among others, antioxidant defenses [12], and activates extracellular signal-regulated kinase [13] and phosphorylation of the cAMP response element-binding protein (CREB) [14]. The activation of CREB results in the expression of pro-survival genes such as those encoding brain-derived neurotrophic factor (BDNF) [15,16] or its receptor, tropomyosin-related kinase B (TrkB) [17]. In contrast, activation of NMDARs in pathological conditions—for example, after massive glutamate release in the ischemic brain—opposes these neuroprotective effects and is coupled to cell death pathways [18]. In stroke, deprivation of oxygen and glucose causes an important decrease in ATP levels, followed by membrane depolarization and the release of excitatory neurotransmitters that induce NMDAR overactivation, massive Ca^2+^-influx and excitotoxicity. Different mechanisms participate in this form of neuronal death, but a major contribution is made by calpain, a Ca^2+^-dependent protease central to different acute and chronic CNS disorders associated with excitotoxicity [19]. In neurons, calpain processes many different substrates in discreate specific sequences altering their stability, activity or localization [20,21]. Importantly, some of these calpain substrates are proteins critical to neuronal survival and function, such as the neurotrophin receptor TrkB [22], the GluN2 subunits of the NMDAR [23] or their interacting protein PSD-95 [23,24].

The dual roles played by NMDARs in neurons have been related to their cellular location, whereas synaptic NMDARs mediate survival signaling pathways, and extrasynaptic receptors activate death pathways. However, another theory states that GluN2A-containing NMDARs are implicated in neuronal survival, while GluN2B-containing NMDARs induce neuronal death [25]. Actually, the “NMDAR location” and “NMDAR subtype” hypotheses are not mutually exclusive since, in the adult cortex, GluN2A and GluN2B subunits are preferentially localized at, respectively, synaptic and extrasynaptic sites. Nevertheless, recent studies have pointed out that the activation of death signaling pathways requires the synergistic activation of both synaptic and extrasynaptic NMDARs [25]. Although we still need a more in depth characterization, our current knowledge about the dual roles of NMDARs in physiopathology has helped to understand previous failure of NMDAR antagonists in stroke clinical trials. This knowledge has also provided solid ground for the development of novel neuroprotective strategies for stroke that are able to inhibit death signaling and downstream NMDAR overactivation, without interfering with survival or, alternatively, strategies aimed at preventing aberrant downregulation of neuronal survival cascades taking place in excitotoxicity. Since PSD-95 plays a central role in neuronal survival and death downstream NMDARs, it has become a very promising target for both types of therapeutic strategies. Such therapies might protect and recover the ischemic penumbra, contributing to reduced stroke damage, but the therapy could be also used to treat other acute and chronic diseases, such as neurodegenerative diseases, which are similarly associated with excitotoxicity [26].

## 3. PSD-95, a Protein Central to Neuronal Survival and Death

### 3.1. PSD-95 Structure and Function

**Figure 1:**
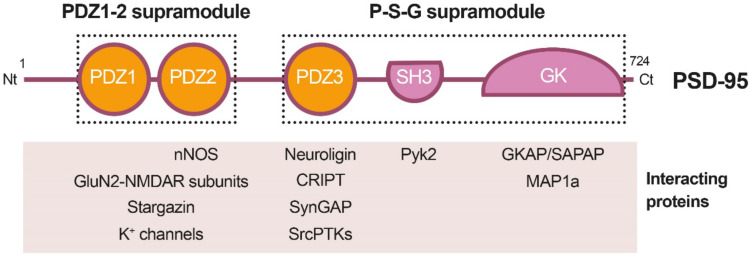
Diagram of PSD-95 protein domains, their organization in supramodules and some examples of their interacting proteins. From the N-terminal (Nt) to the C-terminal (Ct), PSD-95 is constituted by three PDZ (PDZ1 to 3) domains, a Src homology 3 (SH3) domain and a guanylate kinase-like (GK) domain. The PDZ1-2 and P-S-G supramodules, respectively formed by the PDZ1 and PDZ2 tandem or the three other protein domains, are indicated by dotted outlined rectangles.

**Figure 2:**
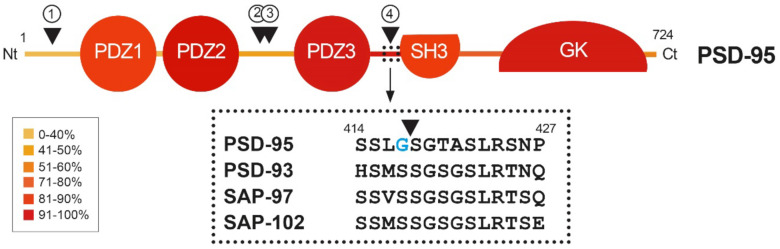
Heatmap showing amino acid conservation inside the different protein domains and interdomain linker sequences of DLG proteins. The location of four calpain-cleavage sites established by Edman sequencing in PSD-95 is also shown (numbered arrowheads). Mice protein sequences of PSD-95, PSD-93, SAP-97 and SAP-102 were compared using Clustal Omega. The percentage of conservation was calculated including identical amino acids and conservative changes, and a color code was assigned to each protein segment. The different DLG domains present a high level of conservation (70–90%), while linkers between them are less conserved, except for the PDZ3–SH3 sequence (94%). Notably, PSD-95 is processed by calpain inside this highly conserved sequence (arrowhead), while other DLG members are not. Substitution of S417 for G417 (blue) inside this highly conserved sequence of PSD-95 might have been determinant for creating a calpain-specific recognition sequence and cleavage of this particular DLG protein in excitotoxic conditions.

PSD-95 is a member of the Discs large (DLG) family of scaffold proteins, which are a class of the membrane-associated guanylate kinases (MAGUKs), important in cell–cell adhesion and regulation of receptor functioning and clustering [27]. Thus, many of these proteins can interact with the NMDARs through specific protein–protein interaction domains, known as PSD-95/Disc large/Zonula occludens-1 (PDZ) domains. Regarding PSD-95 as a major component of the PSD, it shapes a framework of multiple proteins at excitatory synapses [28] (Figure 1) that organizes signal transduction and is central to glutamatergic synaptic signaling [27]. It is structurally organized in different protein domains (Figure 1) containing, from the N-terminus, three PDZ domains (PDZ1–PDZ3) followed by a Src homology 3 (SH3) domain and a guanylate kinase-like (GK) domain, all of them highly conserved (Figure 2). The PDZ domains interact directly with the C-termini of NMDAR-GluN2 subunits (known as PDZ-ligands or PDZ-binding domains), among others, or they interact indirectly with α-amino-3-hydroxy-5-methyl-4-isoxazolepropionic acid receptors (AMPARs) through PDZ-ligands present in AMPAR auxiliary proteins such as stargazin/transmembrane AMPAR regulatory proteins (TARPs). These indirect PDZ interactions, by regulating the content of AMPARs at dendritic spines, are essential for basal synaptic transmission and establishment of long-term potentiation (LTP) [29].

Recent work has suggested that PSD-95, as well as other related proteins, is functionally and structurally organized in two supramodules that result in a supertertiary structure [30]. The first PSD-95 supramodule contains the PDZ1 and PDZ2 domains, and is known as the PDZ1-2 supramodule (Figure 1). PDZ orientation is restrained in this supramodule but changes to a more flexible conformation, with enhanced binding affinity, upon interaction with PDZ-binding domains of interacting proteins [31]. A very important PDZ1-2 function is the assembly of a ternary complex with the NMDAR-GluN2B subunits and nNOS, established by interaction of the GluN2B PDZ-ligand with PSD-95 PDZ1 or PDZ2, and a noncanonical insertion of a nNOS β-hairpin motif in the binding pocket of PDZ2 [32]. This ternary complex is central to neuronal physiological functions, including plasticity, learning or memory. However, in an excitotoxic context, this complex promotes the coupling of NMDAR overstimulation and Ca^2+^ influx to nNOS activation and production of nitric oxide (NO), which can react with cell superoxide free radicals, forming oxidant peroxynitrite. This is a highly reactive molecule that produces oxidation of proteins and lipids and generates DNA damage, leading to the activation of apoptotic factors and apoptotic death [33]. In addition to mediating NMDAR-dependent excitotoxicity, NO also negatively regulates the fate of neural stem cells (NSCs) and inhibits regenerative repair after stroke in rat models [34].

Domains PDZ3, SH3 and GK form the second PSD-95 supramodule, known as the P-S-G supramodule, where PDZ3 directly couples with the SH3–GK tandem in a PDZ-ligand binding-dependent manner [35]. Thus, PDZ3 interaction with cysteine-rich PDZ-binding protein (CRIPT) induces a conformational modification of the SH3–GK domain, which influences subsequent formation of PSD-95 homotypic and heterotypic complexes [36,37], and binding of the heterotrimeric G protein subunit Gnb5 to PSD-95 GK domain at dendritic spines [38]. An additional function of the PDZ3 interaction with CRIPT, a protein that also binds microtubules, is PSD-95 linkage to neuronal cytoskeleton and regulation of dendritic arborization and spine number [39]. Altogether, these results reveal that the organization of the PSD-95 complexes is guided by hierarchical binding mechanisms.

### 3.2. Regulation of PSD-95 Location and Expression

Given the importance of PSD-95 in PSD formation, and neuronal function and survival, this protein location and expression are highly regulated in neurons. Different posttranslational mechanisms regulate PSD-95 location at the PSD [40]. This protein is associated with the PSD membrane in a perpendicular way through its N-terminus, the C-terminus being oriented to the dendritic spine. A major mechanism regulating this location is the palmytoilation/depalmytoilation of PSD-95 residues C3 and C5, proposed to facilitate the formation and stabilization in development of postsynaptic membrane microdomains that might be central to the organization and function of neuronal synapses [41]. Additional protein modifications such as phosphorylation, ubiquitination, nitrosylation, and neddylation also regulate PSD-95 synaptic stability, protein interactions and function [40,42].

Concerning PSD-95 levels, this protein increases in normal cortex from early life, reaching maximum levels during adolescence or early adulthood [43], suggesting that this rise might be relevant to synaptic maturation. Moreover, disruption of PSD-95 expression has been associated with synaptic dysfunction in neurodevelopmental disorders such as schizophrenia [44,45] or autism [46]. Another example of the relevance of PSD-95 levels is the observation that combined knockdown of PSD-95, PSD-93 and SAP-102 reduces synaptic transmission regulated by AMPARs and NMDARs and alters PSD size and structure [47]. Additionally, an acute depletion of PSD-95 levels induces death of hippocampal neurons through activation of an alpha Ca^2+^/calmodulin-dependent protein kinase II (αCaMKII) transduction pathway [48]. Other studies have also shown that a genetic deletion of the GK domain unpredictably inhibits PSD-95 expression. In neurons, PSD-95 forms a ternary protein complex with both the D_1_ dopamine receptor and NMDARs that limits their direct interaction and dampens reciprocal receptor potentiation. Thus, neurons lacking PSD-95 are more susceptible to NMDAR-mediated excitotoxicity, which causes neuronal damage and neurological impairment [49]. Furthermore, it has been recently demonstrated that PSD-95 protects synapses from ß-amyloid toxicity, suggesting that low levels of synaptic PSD-95 might be a molecular sign indicating synapse vulnerability to Aß in Alzheimer’s disease [50].

### 3.3. PSD-95 Downregulation in Ischemia Models

**Figure 3:**
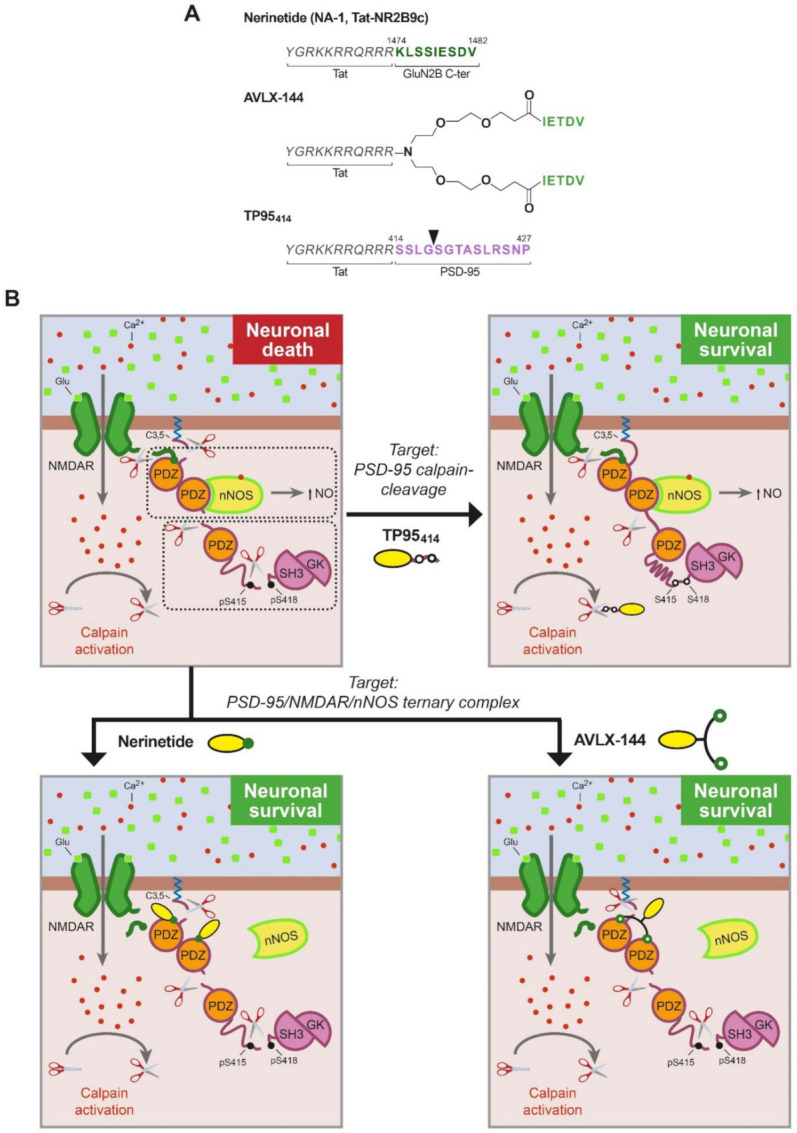
(**A**). Structure of PSD-95-targeted peptides for stroke treatment. The three peptides share a Tat sequence (aa 47–57, italic). In nerinetide, this sequence is bound to the GluN2B C-terminus (dark green), containing the PDZ ligand, while AVLX-144 is a dimeric peptide with slightly different PDZ ligands (light green) that are more selective towards PDZ1 and PDZ2 over PDZ3. In TP95_414_, the Tat sequence is followed by a PSD-95 sequence (aa 414–427, purple) containing a calpain-cleavage site (arrowhead). (**B**). Model of PSD-95 regulation in excitotoxicity and action of PSD-95-targeted peptides. In excitotoxicity, without treatment, PSD-95 contributes to neuronal death by two different mechanisms: first, formation of a trimeric PSD-95–NMDAR–nNOS complex that produces nitric oxide (NO) upon massive calcium influx, and second, processing by calpain of PSD-95 that uncouples the two PSD-95 supramodules (dotted outlined rectangles) and partially cleaves P-S-G. Two different strategies have been developed to prevent these pathological mechanisms by means of brain-permeable neuroprotective peptides. Nerinetide and AVLX-144 are targeted to uncouple the trimeric complex and inhibit NO production while TP95_414_ prevents PSD-95 cleavage.

A dramatic decrease in PSD-95 levels has been also unveiled as an important pathological mechanism contributing to neuronal death in models of in vitro excitotoxicity, transient ischemia induced in rat by middle cerebral artery occlusion (tMCAO) [23] or permanent ischemia induced by microvascular photothrombosis in mice [24]. PSD-95 downregulation is induced very early after permanent stroke, the complete protein being hardly detectable 2.5 h after injury, and this decrease correlates with the progressive formation of stable protein fragments. Similar PSD-95 fragments are produced in primary cultures of cortical neurons after NMDAR overactivation, while other DLG proteins such as SAP-97 or SAP-102 are not processed in these excitotoxic conditions [24]. The major mechanism responsible for PSD-95 downregulation in excitotoxic neurons is the activation of calpain as a consequence of Ca^2+^-influx due to NMDAR overactivation (Figure 3B). In fact, PSD-95 is a calpain substrate that is cleaved by this protease in four specific sequences, precisely established by Edman sequencing of protein fragments produced in vitro by digestion with purified enzyme (Figure 2, arrowheads). Interestingly, the four identified cleavage sites are inside interdomain linker sequences that, among the DLG proteins, are generally less conserved than the protein-interaction domains (Figure 2, heatmap). An exception is the PDZ3–SH3 linker, a very flexible sequence described as critical for P-S-G supertertiary structure [30], which presents a very high homology (94%). This sequence mediates a weak and dynamic PDZ3–SH3 association that, as mentioned before, can be reverted by binding of PDZ-ligands to PDZ3 or electrostatic repulsion due to protein phosphorylation. Notably, PSD-95 is processed by calpain between amino acids G417 and S418 inside this highly conserved sequence. Substitution of S417 for G417 in PSD-95 (Figure 2, blue) might have been a determinant for creating a calpain-specific recognition sequence for this particular DLG protein, absent in the other family members. Remarkably, the result of PSD-95 calpain processing induced by excitotoxicity and stroke is the separation of the PDZ1–2 and P-S-G supramodules, together with partial truncation of the latter to uncouple the PDZ3 and the SH3–GK tandem (Figure 3B) [24]. The effects of PSD-95 cleavage on neuronal survival and functioning will be probably derived from rupture of this protein supertertiary structure and production of a set of stable fragments of yet undefined functions.

## 4. Development of PSD-95-Targeted Cell-Penetrating Peptides for Stroke Treatment

The central role played by PSD-95 in neuronal survival and death makes this protein a very interesting target for the development of neuroprotective strategies for stroke and other pathologies associated to excitotoxicity. Two different approaches targeting PSD-95 are currently under investigation. A very relevant strategy is directed toward preventing the induction of PSD-95-dependent pro-death signaling and neurotoxicity by dissociation of GluN2B–PSD-95–nNOS complexes. However, an important caveat to this approach is PSD-95 calpain-processing, a pathological mechanism that results in a profound alteration of survival signaling. Thus, the stabilization of PSD-95 is being explored as an alternative, and possibly complementary, neuroprotective strategy. Here, we review three very promising cell-penetrating peptides (CPPs) exploiting these neuroprotective strategies that have advanced to different levels of development.

Treatment of CNS pathologies with therapeutic molecules is intrinsically difficult due to the presence of the blood–brain barrier (BBB). An innovative idea at the center of pharmaceutical research is shuttle-mediated delivery of the therapeutic molecules by conjugation to compounds able to cross the BBB, among them, small naturally derived peptides. CPPs are defined as a group of relatively short peptides (4–40 aa) of different chemical characteristics (frequently positive charged) that can get through the BBB and cellular membranes by means of different mechanisms, mainly endocytosis [51]. Moreover, CPPs have the capacity to act as carriers, facilitating the delivery of different types of covalently or non-covalently conjugated bioactive cargoes (e.g., other peptides, small molecules, nucleic acids and even proteins) with very low toxicity. These carrier molecules have a great potential for stroke treatment since brain delivery might be further increased after damage, due to early BBB disruption [52] and stroke promotion of excitotoxicity-induced endocytosis [53], an important cell entry route for CPPs, as mentioned before [51]. One of the most frequently used CPPs is a short 11 aa basic sequence derived from human immunodeficiency virus (HIV) Tat protein, which is responsible for Tat transduction properties. In fact, the three PSD-95-targeted peptides described below share a Tat peptide as carrier molecule (Figure 3A).

### 4.1. Nerinetide, Renewed Hope for Neuroprotection in Human Stroke

The development of nerinetide by Dr. Tymianski’s group has set the standard for how to navigate from basic research to clinical translation in human stroke. Nerinetide, previously known as NA-1 or Tat-NR2B9c, is an eicosapeptide formed by the Tat sequence followed by the 9 C-terminal residues of NMDAR-GluN2B subunit, containing the PDZ-ligand (Figure 3A). Considering the hypothesis that NMDARs formed by GluN2B subunits are predominantly involved in neuronal death signaling, this peptide was designed and shown to dissociate GluN2B–PSD-95–nNOS complexes, uncoupling NMDARs from nNOS neurotoxic signaling and suppressing NO production (Figure 3B) [54]. In contrast, a similar peptide that no longer resembled the GluN2B PDZ-ligand (Tat-NR2B9cAA) was not able to decrease GluN2B–PSD-95 interaction. Remarkably, different from previous treatments with NMDAR antagonists, nerinetide did not block the synaptic activity of NMDARs. Moreover, this peptide (3 nmol/g) was also neuroprotective in rat models of transient or permanent stroke even when administered 3 h after injury, significantly reducing infarct volume and improving the neurobehavioral outcome [54,55,56], reviewed by [10,57]. These results highlighted the potential clinical usefulness of nerinetide and stimulated new studies designed to bridge the gap between rats and humans. Thus, the effects of nerinetide were analyzed in gyrencephalic non-human primates that presented genetic, behavioral and anatomical similarities to humans [58]. Administration of this peptide after the induction of stroke injury was neuroprotective in macaques subjected to MCAO in an experimental context that mimicked clinically relevant conditions. The infarct volume was significantly reduced by nerinetide treatment, and furthermore, the ischemic cells retained their integrity and transcription capacity, in correspondence with the preservation of neurological function in neurobehavioral assays. These results were ground-breaking in the field of stroke neuroprotection and strongly suggested that the strategy of targeting PSD-95 rather than NMDARs might also reduce stroke damage in humans.

A Phase 2 clinical trial assay called ENACT (Evaluating Neuroprotection in Aneurysm Coiling Therapy; ClinicalTrials.gov number, NCT00728182) was completed in 2012. This study was aimed at evaluating the safety and efficacy of nerinetide for reducing embolic stroke in the context of an endovascular treatment of intracranial aneurysms [59]. A double-blind, randomized, controlled study was conducted in Canada and the USA, enrolling close to 200 patients having ruptured or unruptured intracranial aneurysm amenable to endovascular repair. Small embolic ischemic strokes are a very frequent complication of endovascular intracranial aneurysm repair. Patients receiving a single intravenous infusion of nerinetide at the end of the endovascular procedure presented fewer ischemic infarcts as compared with those treated with saline, suggesting that neuroprotection is possible in human ischemic stroke. Thus, the next goal of this group was to investigate this promising strategy in larger clinical trials. Nerinetide has recently completed a Phase 3 clinical trial called ESCAPE-NA-1 (Safety and Efficacy of Nerinetide (NA-1) in Subjects Undergoing Endovascular Thrombectomy for Stroke; ClinicalTrials.gov number, NCT02930018) [7]. The primary objective of this assay was to determine the efficacy and safety of a single intravenous dose of nerinetide in reducing global disability in subjects with AIS due to a large vessel occlusion, within a 12 h treatment window. Recruited patients had a small-to-moderate infarct core and moderate-to-good collateral circulation and were selected for rapid endovascular revascularization. This randomized, multicenter, blinded, placebo-controlled parallel group study enrolled 1105 patients undergoing both thrombolytic or thrombectomy therapies. When globally analyzed, the results showed no significant differences between the nerinetide and the placebo groups. However, a significant effect of nerinetide treatment was observed in patients undergoing endovascular thrombectomy without prior thrombolysis with alteplase (recombinant tPA), a standard-of-care thrombolytic agent. These patients presented improved functional outcome, reduced mortality, and decreased infarction volumes compared with those receiving placebo. These findings suggested a possible drug–drug interaction between nerinetide and alteplase that suppresses the therapeutic effect of nerinetide. In fact, pharmacokinetic data obtained from trial participants demonstrated a decrease in the concentration of nerinetide in patients treated with nerinetide and alteplase, compared with those who did not receive alteplase [7]. Thus, the results of the ESCAPE-NA-1 trial obtained in the subgroup of participants that did not receive alteplase are promising. This approach will be further tested in a Phase 3 trial called ESCAPE-NEXT (ClinicalTrials.gov NCT04462536), currently recruiting patients with arterial ischemic stroke selected for endovascular thrombectomy without the use of a tPA (alteplase, tenecteplase or equivalent).

Nevertheless, the drug–drug interaction between alteplase and nerinetide is still problematic for stroke patients who qualify for alteplase treatment and justifies further investigation. The aminoacidic sequence of nerinetide is susceptible to cleavage by plasmin, a serine protease generated from circulating plasminogen by alteplase [60]. Thus, new ways of improving nerinetide effectiveness are currently under investigation. One possibility for preventing plasmin recognition is the substitution of the cleavage-prone nerinetide amino acids from their L- to their D-enantiomer. When tested in a rat model of embolic MCAO, this new peptide lost plasmin sensitivity while maintaining the neuroprotective effect, even if administered together with alteplase [61]. Alternatively, unmodified nerinetide could maintain its effectiveness when administered before alteplase. Due to the short half-life of this peptide, application of the thrombolytic agent a few minutes after nerinetide infusion was sufficient to completely avoid drug–drug interaction. This approach is currently under evaluation in an ongoing clinical trial called FRONTIER (Field Randomization of NA-1 Therapy in Early Responders; ClinicalTrials.gov NCT02315443), in which patients with suspected stroke are treated with nerinetide or placebo early after symptom onset, on their way to the hospital and before receiving thrombolysis or endovascular thrombectomy. According to previous results [61], alteplase would not interfere with nerinetide under these conditions. Altogether, accumulated knowledge about nerinetide makes this PSD-95 inhibitor a very promising candidate for AIS treatment and brings new hope for neuroprotection in human disease.

### 4.2. AVLX-144, a High-Affinity Dimeric Peptide against Stroke

The dissociation of GluN2B–PSD-95–nNOS complexes, without affecting the physiological functions of the NMDARs, has drawn the interest of other research groups that have developed different compounds having mechanisms of action similar to those of nerinetide. Some of them are small molecule inhibitors targeting the PDZ domains of PSD-95, such as IC87201 or ZL006, recently reviewed elsewhere [62]. The group led by Dr. Strømgaard (University of Copenhagen, Copenhagen, Denmark) has also chosen peptides as therapeutic tools and has conducted excellent work in optimizing the PDZ-ligands for increased affinity, as well as in improving peptide stability and selectivity. Starting from the observation that the five C-terminal amino acids of GluN2B are sufficient to bind the PDZ1 and PDZ2 domains with affinity similar to that of the GluN2B undecapeptide [63], they designed several symmetric dimeric ligands using monodisperse polyethylene glycol (PEG) to produce linkers of different lengths. The affinity of some of these dimers strongly increased relative to that of the monomeric peptide [64]. Another interesting observation was that crosslinking with PEG substantially prolonged peptide stability in plasma. After that, they modified the PEG linker to include a central amine handle in order to attach a Tat peptide, directed at improving the permeability across the BBB [65]. The resulting peptide, AVLX-144 (Figure 3A), previously known as UCCB01-144, is a high-affinity dimeric ligand towards PDZ1–PDZ2 (Figure 3B). In fact, compared with the monomeric peptide nerinetide, AVLX-144 has a 1000-fold increase in affinity, which is directly related to its dimeric nature. Analysis by NMR and small-angle X-ray scattering (SAXS) showed that the interaction of the dimeric ligand with PDZ1-2 led to a more extended and flexible conformation than unbound PDZ1-2 but more compact and restricted compared with the PDZ1–2 in complex with the monomeric ligand [65].

Using a mouse model of permanent MCAO, they demonstrated that treatment with an intravenous dose of AVLX-144 (3 nmol/g) 30 min after ischemia protected against brain damage and improved neuromuscular function. Interestingly, nerinetide did not show significant effects in this mice model when used at this concentration [65], but it significantly reduced infarct size at a higher dose (10 nmol/g) [66]. Thus, nerinetide seems to be less effective in mice as compared with rat models of stroke [65,66]. To deepen these results, the dose–response relationship of AVLX-144 and nerinetide was investigated in the same mice model. Both peptides decreased infarct size to a similar extent when used at 9 nmol/g, confirming previous results, but at higher doses (30 nmol/g) AVLX-144 was not neuroprotective, while nerinetide produced acute toxicity (28 out of 30 mice died immediately after injection) [67]. In order to address potential adverse cardiovascular effects of treatment with high peptide doses of AVLX-144 or nerinetide, they next measured systemic blood pressure and heart rate in non-ischemic mice. No significant differences were found in blood pressure for the different concentrations employed (3, 9 or 30 nmol/g) compared with saline. Regarding heart rate, no alterations were found for 3 or 9 nmol/g, while significant differences were observed for both peptides at the highest dose. While AVLX-144 induced an increase in heart rate around 80 min after peptide administration, nerinetide produced a dramatic drop immediately after injection, and the heart rate did not return to normal levels until 20–30 additional minutes [67]. These side effects of nerinetide might explain why the majority of the ischemic mice, more vulnerable than non-ischemic mice, died after injection of high peptide doses. Altogether, these results suggest that the effective therapeutic window is narrower for nerinetide than for AVLX-144, at least in this mouse model of permanent MCAO. This group has also examined the effect of biological variation on neuroprotection by AVLX-144. Using the same ischemia model, they compared previous results, obtained with young adult mice, with aged male mice (12 months) or young female mice [67]. They observed that AVLX-144 (9 nmol/g) reduced infarct size in aged male mice, similar to young animals, but was not effective in female mice, even after lowering estradiol levels. These results convey the need to perform more preclinical assays to elucidate the sex-specific neuroprotective effects of the PSD-95 inhibitors.

The potential of AVLX-144 as neuroprotectant has encouraged further studies towards clinical translation. A Phase 1 study in healthy volunteers to evaluate safety, tolerability and pharmacokinetics is currently underway (ClinicalTrials.gov, NCT04689035). This is a randomized, double-blind, placebo-controlled, single ascending dose study that plans to first analyze AVLX-144 safety and tolerability in healthy male subjects (age 20–50 years old). Once establishing the drug dose, healthy elderly subjects (65–80 years of age, both males and females) will be analyzed.

### 4.3. TP95_414_, a Novel Neuroprotective Approach for Preventing PSD-95 Processing

The second approach to stroke treatment targeted to PSD-95 is the prevention of the characteristic processing of this protein induced by NMDAR overactivation. A previous work demonstrated that it is possible to design CPPs that interfere with the processing of a specific calpain-substrate in excitotoxicity and produce stroke neuroprotection [68]. Thus, a Tat-based CPP (TFL_457_) containing TrkB-FL sequences controlling calpain-processing of this neurotrophin receptor was developed and shown to sustain survival signaling in excitotoxic conditions. This neuroprotective peptide decreased the infarct size and improved the neurological outcome in a mice model of permanent stroke [68]. Therefore, it was reasonable to propose that interference of PSD-95 processing and maintenance of PSD-95 levels could preserve the structure and function of this scaffold protein and constitute a novel therapeutic strategy for stroke treatment. As mentioned before, the combination of biochemical analysis, in silico studies and fragment purification and sequencing identified four cleavage sites between PSD-95 positions 33–34, 259–260, 279–280 and 417–418 (Figure 2) [24]. This information allowed the rational design of three Tat-derived peptides (TP95_29_, TP95_259_ and TP95_414_) containing the PSD-95 calpain-cleavage sequences that potentially could specifically interfere with this processing. This strategy, aimed at preventing the pathological processing of this specific calpain substrate, is not expected to interfere with the physiological functions of PSD-95. The use of generic calpain inhibitors as an alternative strategy was ruled out because they might inhibit the physiological actions of this protease and produce unwanted side effects [10].

Only one of the generated PSD-95 peptides, TP95_414_, containing PSD-95 residues 414–427 (Figure 3A), was able to preserve neuronal viability and morphology in primary culture of rat cortical neurons subjected to excitotoxicity, a result that correlated with PSD-95 stabilization. A similar Tat peptide containing unrelated sequences, previously shown to enter neurons without affecting neuronal viability in basal or excitotoxic conditions [68], did not have these neuroprotective effects. It is interesting that TP95_414_, which compared with peptides TP95_29_ and TP95_259_ contains the PSD-95 sequence less efficiently processed in vivo, could not only partially preserve the P-S-G supramodule but also the complete protein (Figure 3B). Results also suggest that calpain-processing at this PSD-95 site (G417/S418) might be regulated by phosphorylation [24], a protein modification that could make these sequences at the PDZ3-SH3 linker more accessible for calpain. In fact, it has been described that phosphorylation of residues S415 and S418 increases PDZ3 mobility relative to the SH3–GK tandem and disrupts the interactions of these domains [69]. One possibility is that regulated cleavage at G417/S418, inside the P-S-G supramodule, might determine further PSD-95 processing in the three other established calpain-processing sequences surrounding the PDZ1–2 supramodule.

Further studies have demonstrated that peptide TP95_414_ can cross the BBB and reach the cortex, where it is found in cell bodies and neurites of cortical neurons. Using a severe mice model of permanent ischemia, where a narrow ischemic penumbra [70] defies the efficacy of neuroprotective molecules, the administration of TP95_414_ (3 nmol/g) 10 min after injury tends to interfere with PSD-95 processing and decrease infarct volume. Despite this modest decrease in infarct volume (15%), TP95_414_ significantly improves the neurological function of treated animals. It is interesting that no effects were observed for a peptide concentration of 10 nmol/g, a result supporting the importance of dose–response experiments and suggesting that levels of PSD-95 have to be maintained in a correct range to support neuronal survival after brain damage.

Altogether, these results unveil PSD-95 stabilization as an attractive and highly relevant target for stroke therapy and perhaps for other important neurological conditions different from stroke, likewise associated with excitotoxicity.

## 5. The Quest for Specificity in Stroke Therapies Based on PSD-95-Targeted Peptides

The development of the neuroprotective peptides reviewed here has been guided by a compromise with rationally designed molecules that are able to challenge specific pathological mechanisms activated in neurological diseases, stroke included. We have learned very important lessons from the past use of antagonists of glutamate receptors for stroke treatment. They lacked specificity, and thus, their administration affected both physiological and pathological receptor functions, producing unwanted side effects. Therefore, efforts in the field of neuroprotection have focused on the development of brain accessible molecules able to specifically target proteins or mechanisms downstream of NMDAR overactivation related to the pathological state. A thorough delineation of the dual roles of NMDARs in neurons has identified critical downstream death-signaling pathways that can be specifically inhibited without blocking the NMDARs [10,25]. These therapeutic approaches, as well as those reviewed here, have required strong mechanistic insights about the disease-specific pathophysiology.

Many research groups have chosen CPPs to deliver specific therapeutic molecules to the CNS, and some of these neuroprotective peptides, as mentioned before, have reached clinical trials. Several other specifically targeted peptides are currently being tested in preclinical models of disease and have great translational potential. In a different approach, cationic arginine-rich peptides have been proposed as neuroprotective agents for stroke and other excitotoxicity-associated disorders. Thus, it has been recently shown that poly-arginine peptide-18 (R18) induces a decrease in brain injury and modestly improves functional outcome in a nonhuman primate stroke model [71]. It has been described that these non-targeted CPPs have a multimodal mechanism of action. In addition to their ability to cross cell membranes and protect neurons from glutamate excitotoxicity and intracellular Ca^2+^-influx, these peptides reduce neuronal surface expression of NMDAR subunits and the activity and/or surface expression of other ion channels and receptors. It has been also suggested that they target mitochondria, stabilize proteins, inhibit proteolytic enzymes, induce pro-survival signaling, scavenge toxic molecules and reduce oxidative stress [72]. Such a complex mechanism of action needs to be carefully considered before evaluating their translational potential. Treatment with these peptides might affect multiple critical aspects of neuronal function, both in damaged and undamaged cells alike, and thus could have adverse effects on brain function.

## 6. Potential of Combined Approaches for Stroke Neuroprotection Using PSD-95 Targets

We have summarized here results unveiling two different approaches for stroke neuroprotection directed at PSD-95: uncoupling of the NMDAR–PSD-95–nNOS complex and prevention of PSD-95 downregulation. Treatment with nerinetide or AVLX-144 alone will inhibit pro-death pathways activated by coupling of the NMDARs and nNOS in stroke but would not affect PSD-95 processing by calpain, still leading to profound changes in PSD and synapses (Figure 3B). Additionally, after TP95_414_ treatment, PSD-95 levels can be maintained in the damaged brain, but the formation of the trimeric complex and NO production would not be inhibited, thus limiting the therapeutic effect of this neuroprotective peptide. Therefore, we propose that the combination of both strategies could result in a more powerful neuroprotective effect since promotion of PSD-dependent pro-survival pathways and inhibition of pro-death actions would take place at the same time. This combined neuroprotective approach could be highly relevant for stroke therapy.

## 7. Conclusions

In this article, we reviewed evidence demonstrating that PSD-95 is a promising target for ischemic stroke therapy, as well as for other CNS disorders. The usage of CPPs able to block PSD-95 pro-death functions and/or favor the maintenance of this protein’s pro-survival activities during excitotoxicity have a great potential for neuroprotection.
